# Error in the Byline

**DOI:** 10.1001/jamanetworkopen.2023.29097

**Published:** 2023-08-01

In the Invited Commentary titled “Elucidating the Underlying Mechanisms of the Marked Increase in Childhood Type 1 Diabetes During the COVID-19 Pandemic—The Diabetes Pandemic,”^1^ published June 30, 2023, there is a missing letter in Dr Kamrath’s name in the byline. This article has been corrected.^1^
